# Opening threshold and kinetics of the MscL mechanosensitive channel are regulated by its periplasmic loop

**DOI:** 10.21203/rs.3.rs-9216610/v1

**Published:** 2026-05-05

**Authors:** Kingsley C. Duru, Paul R. Rohde, Hooman Hafezi, Omid Bavi, Navid Bavi, Boris Martinac

**Affiliations:** Victor Chang Cardiac Research Institute; Victor Chang Cardiac Research Institute; Shiraz University of Technology; Shiraz University of Technology; University of California, Los Angeles; Victor Chang Cardiac Research Institute

**Keywords:** Patch clamp, EPR spectroscopy, giant E. coli spheroplasts, site-directed mutagenesis, liposomes, molecular dynamics simulations

## Abstract

Mechanosensitive (MS) channels are membrane proteins that respond to mechanical stimuli and are essential across prokaryotic and eukaryotic organisms. The *E. coli* mechanosensitive channel of large conductance (MscL) provides a powerful model for dissecting protein–lipid interactions underlying mechanotransduction. Here, we investigated the contribution of four periplasmic loop residues (A64, Q65, G66, D67) to MscL gating. Using site-directed spin labelling (SDSL) and electron paramagnetic resonance (EPR) spectroscopy, we show that these residues interact directly with the lipid bilayer during the channel opening through membrane tension sensitivity of the channel. We further examined how mutations at these periplasmic loop residues affect biophysical properties of MscL in giant *E. coli* spheroplasts and liposomes composed of azolectin or negatively charged lipids (70% phosphatidylcholine and 30% phosphatidylglycerol) using patch clamp electrophysiology, patch fluorometry and molecular dynamics simulations. Substitution of Q65 residue with arginine (Q65R) increased channel sensitivity to membrane tension, whereas replacement with glutamic acid (Q65E) decreased the sensitivity across all systems tested. Molecular dynamics simulations revealed that under in-plane radial tension Q65E exhibited larger conformational changes under tension, whereas the wild-type (WT) and Q65R mutant channels showed rapid and extensive opening at later stages within a shorter time frame. Insertion of a four-glycine hinge at D67 also reduced tension sensitivity, consistent with impaired force transmission. Substitution of A64 and G66 to either glutamic acid (A64E, G66E) or arginine (A64R) decreased tension sensitivity of the channel only in liposomes of negatively charged lipids. Together, these findings deepen our understanding of protein-lipid interactions governing MscL opening kinetics and highlights the contribution of the periplasmic loop in regulating mechanosensitivity.

## Introduction

Mechanosensitive (MS) channels are a diverse class of membrane proteins found in prokaryotes and eukaryotes that are gated in response to mechanical stimuli and play critical roles in touch and sound sensation and in cardiovascular mechanotransduction ^1, 2, 3, 4, 5, 6, 7, 8^. In prokaryotes, they play a crucial role in osmoregulation, preventing cell lysis during acute hypo-osmotic shocks ^9^. Bacterial MS channels, including the mechanosensitive channels of large and small conductance, MscL and MscS, respectively, have been found to respond to membrane tension transmitted solely through the membrane bilayer without the need for any cytoskeletal components for the transmission of the mechanical force ^10, 11, 1^. Additionally, membrane curvature, including that induced by inserting amphipathic compounds into a single leaflet of the bilayer, can activate bacterial MS channels ^12, 13^. This makes them an excellent model to dissect protein-lipid interactions underlying mechanotransduction ^14^.

MscL, which is the first cloned MS channel, has a large nonselective pore (~30 Å in diameter) that allows for the passage of ions, solutes and even small proteins, thus preventing cell lysis ^15, 16, 17^. This pore feature also makes MscL a potential pharmacological target in drug-resistant bacteria ^18, 19, 20^. The crystal structure of the *Mycobacterium tuberculosis* MscL (TbMscL) showed that it forms a homopentamer, with each monomer consisting of a relatively short N terminus, two transmembrane α-helices, TM1 and TM2 that are connected by a periplasmic loop, and a cytoplasmic C-terminal helix ^21, 22, 23^. Patch-clamp recordings utilising mutant MscL channels have elucidated contributions of the amphipathic N terminus, the highly conserved TM1 helix, the periplasmic loop, and the interaction between the TM1 and TM2 helices to channel function and gating mechanism ^24, 25, 12, 26, 27^. Several studies showed that mutations that affect the MscL opening probability also affected bacterial cell growth ^28, 24, 25, 29, 9, 30, 31^. The periplasmic loop, which consists of 29 amino acids starting from G46 and ending at H74 in *E. coli* MscL, has been proposed to contribute to the mechanosensitivity of the channel by stabilizing the closed state and by setting the energy barrier needed to open the channel ^32^. To date only a few studies have addressed a potential role/contribution of this domain to the MscL gating mechanism. One study reported that residue Q56 in the periplasmic loop of *E. coli* MscL contributes both to the mechanosensitivity and gating kinetics of the channel ^28^, while molecular dynamics (MD) simulations suggested that Q65 may interact with lipid headgroups when MscL is in the open conformation ^33^. Furthermore, a large-scale mutagenesis study showed that substitution of Q65 with leucine (Q65L) decreased the tension sensitivity of MscL ^34^, while a subsequent study combining site-directed spin labelling (SDSL) with electron paramagnetic resonance (EPR) spectroscopy showed that substituting Q65 with arginine (Q65R) increased its pressure sensitivity ^35^.

Our initial SDSL EPR study of residues in the periplasmic loop showing that Q65R mutation decreased the mobility of this residue and reduced the mechanosensitivity of the mutant MscL^35^. It also suggested that residues Ala64 (A64), Gly66 (G66), and Asp67 (D67) could also contribute to the mechanosensitivity of MscL given the results of the follow-up SDS EPR study examining the mobility and accessibility of all periplasmic loop residues (Fig. 2a & b). Based on these findings, we decided to explore in more detail the contributions of the four periplasmic loop residues to the structural dynamics of MscL by substituting these residues with glutamic acid (E) or arginine (R) or inserting a four-glycine (4G) flexion point at D67 (D67 + 4G) for minor decoupling to TM2 **(Table S1)**. We applied patch-clamp and patch fluorometry techniques to giant spheroplasts prepared from *E. coli* expressing MscL mutants as well as to proteoliposomes made of different types of lipids containing the mutant channels. We complemented these electrophysiology experiments with MD simulations.

## Materials and Methods

### Clone construction

For MscL expression in *E. coli* used to generate giant spheroplasts, native MscL coding sequence followed by two TAA stop codons (before any silent purification fusion tags), was cloned into pBAD24 vector utilising a pET vector ribosomal binding site, or into the pQE-70 expression plasmid (Qiagen). Mutations were generated by the “QuikChange” site-directed mutagenesis strategy to the required codons whilst also conforming to optimal codon usage for *E. coli* translation. Clones needed for MscL protein production were generated from the pQE clones by deleting the double stop codons by the QuikChange method, to allow the translation of a C-terminal fusion tag coding for GlySerHisHisHisHisHisHis followed by two TAA stop codons. All clones were screened by Sanger sequencing across the coding region, also with variant analysis (DNAStar Seqman Pro), to detect any hybrid mixed clones.

### Preparation of giant spheroplasts

A colony of *E. coli strain* MJF612 (Frag1, ΔMscS, ΔMscK, ΔMscL, ΔYbdG) or *E. coli* strain AW737KO (MscL::CmR knock-out strain) transformed with the appropriate pBAD24 expression plasmid or the AW737KO strain transformed with the appropriate pQE-70 expression plasmid (Qiagen) and with the pREP4 repressor plasmid (Qiagen) was used to inoculate 50 ml LB medium with 100 μg/ml ampicillin and 6.25 μg/ml kanamycin (if the pREP4 repressor vector was used) and grown overnight at 37°C, 90 RPM (2 inch stroke),in a tissue paper capped 250-ml flask). The next morning, 250 μl of the culture was used to inoculate fresh medium and grown to an OD600 of 0.4. To maintain equal OD_600_ between cultures, a variant amount of around 6 ml of this exponential growth culture was transferred to 54 ml fresh medium (same continuing conditions, ampicillin only) with 60 μg/ml cephalexin (Sigma C4895) for septation-inhibited elongation growth. From visual observation, at a time allowing for adequate elongation (≤ 2 hr), 0.02% final L-(+)-arabinose (Sigma-Aldrich A3256) (for pBAD vectors) or 1 mM IPTG (for pQE vectors) was added for expression induction for 20 min, followed by rapid cooling on ice and then storage at 4°C overnight. Cells were pelleted at 2000×RCF, resuspended by swirling in 2.5 ml 0.8 M sucrose (Sigma-Aldrich SO389), pelleted, supernatant removed, and resuspended in 2.5 ml sucrose again. In sequential order with mixing (final concentrations given) the following were added: 50.5 mM Tris pH 7.2 (Sigma-Aldrich T6066), 202 μg/ml lysozyme (Sigma L6876), 84.2 μg/ml DNase I (Sigma DN25), and 6.3 mM EDTA (Chem-Supply EA023). Several minutes of room-temperature incubation was continued until, by visual observation, an adequate size or number of giant spheroplasts were produced optimally before excess degradation. The reaction was halted with Stop Solution (0.665 M sucrose, 19 mM MgCl2, 59.4 mM Tris pH 7.2) and samples were placed on ice. 4 ml of Storage Buffer (0.784 M sucrose, 9.8 mM MgCl2, 9.8 mM Tris pH 7.2) was added, and samples were split to single-use 150 μl aliquots for storage at −20°C. An aliquot was placed on ice to use at the patching rig.

### Protein expression and purification

A colony of *E.coli* strain AW737KO (MscL::CmR knock-out strain) transformed with the appropriate pQE-70 expression plasmid (Qiagen) for MscL-6xHis (C-terminal) expression, and pREP4 repressor plasmid (Qiagen) was used to inoculate 20 ml LB (with 100 μg/μl ampicillin, 11.25 μg/ml kanamycin, and 6.25 μg/ml chloramphenicol) for an overnight culture (at 37°C, 143 RPM (2 inch stroke), tissue paper capped 50 ml tube). Next morning, 10 ml of this culture inoculated 1 L fresh media in 2.8 L flasks (same conditions) and then induced with 1 mM IPTG when the culture reached OD_600_ of 0.8, and then grown for a further 3 hrs. Cells were pelleted (7500 ×g, 15 min, 4°C) and resuspended (in 30 ml chilled 50 mM NaH_2_PO_4_, 100 mM NaCl, 2 mM MgSO_4_, 146 mM sucrose) with a 2–3 mg “sprinkle” of DNase I (Sigma, DN25) and 0.2 mg/ml PMSF (Sigma, P7626), for two rounds of French pressing (chilled) at 18,000 PSI. Debris was pelleted (12,000 ×g, 15 min, 4°C), followed by supernatant centrifugation (215,000 ×g, 2 hr 50 min, 4°C) to yield a membrane fraction pellet that was solubilised in 23 ml PBS, 6.26 mM DDM (Anatrace, D310) overnight at 4°C. After further debris removal (124,000 ×g, 20 min, 4°C), the supernatant was incubated (2.5 hrs, 4°C) with Talon^®^ Metal Affinity Resin (Takara-bio, 635503) that had been washed twice with 10 ml PBS and equilibrated with Wash Buffer (PBS with 1 mM DDM, 5 mM imidazole (Sigma-Aldrich 56750), pH 6.0) for 1 hr at 4°C. The resin was then drained within a 25 ml column (Bio-Rad 7321010), followed by 50 ml Wash Buffer, and eluted with 15 ml 0.5 M imidazole 1.5 mM DDM PBS, pH 6.0 into a 15 ml 100 kDa Amicon^®^ filter concentrator (Merck, UFC910024). After an overnight gravity filtration at 4°C, the concentrate was diluted with 5 ml PBS 1.5 mM DDM, and concentrated by centrifugation to yield a final sample near 500 μl of purified protein, to be stored at 4°C.

### Protein reconstitution into liposomes

Proteins were reconstituted in azolectin lipid or a lipid mixture of 70% phosphatidylcholine (PC18) and 30% phosphatidylglycerol (PG18) using the dehydration/rehydration (D/R) method as previously described ^12^. Briefly, chloroform was used to dissolve lipids before drying under nitrogen to create a fine lipid film. The lipid film was then resuspended in D/R buffer (200 mM KCl, 5 mM HEPES, adjusted to pH 7.2 with KOH), vortexed and sonicated for 15 mins before the addition of the MscL protein at a protein-to-lipid concentration of 1:250 (w/w). The mixture was then allowed to incubate at room temperature for 1 h under gentle rotation before being transferred to overnight incubation at 4°C. Afterwards, the protein-lipid mixture was washed with bio-beads, and the proteoliposomes were collected after ultracentrifugation. The proteoliposomes were resuspended in D/R buffer, spotted onto glass slides, and dehydrated overnight (vacuum desiccator, RT °C) and stored at −20 °C until when used. Prior to electrophysiological study, proteoliposomes were rehydrated with D/R buffer drops, and incubated overnight at 4°C within a petri dish with a moistened blotting paper base to negate evaporation. In some experiments, MscS channel proteins were co-reconstituted with MscL proteins at the same protein-to-lipid ratio.

### Electrophysiological recording

The effects of the MscL mutations on the channel activities were examined in liposomes and *E. coli* giant spheroplasts. The acquisition of data was done at a sampling rate of 5 kHz with 1-kHz filtration, and currents were amplified with an AxoPatch 200B amplifier (Axon Instruments). A symmetric bath and pipette solution was used in the experiments; for liposomes: the bath and pipette solution consisted of 200 mM KCl, 40 mM MgCl2, and 5 mM HEPES, while the bath solution for the spheroplast experiments contained 250 mM KCl, 90 mM MgCl_2_, and 5 mM HEPES (pH 7.2 adjusted with KOH).

### Patch Fluorometry

Wild-type and mutant MscL protein was reconstituted into azolectin liposomes (99.9% wt/wt) with addition of 0.1% rhodamine-PE18:1 at a protein: lipid ratio (wt/wt) of 1:500 as described above. Using an inverted confocal microscope (LSM 700; Carl Zeiss) equipped with a water immersion objective lens (×63, NA1.15), excised patches of the fluorescent labelled proteoliposomes were excited with a 555-nm laser. The pipette tip was bent ~28° with a microforge (MF-900; Narishige) to align parallel with the bottom face of the recording chamber to enhance visualization of patches ^36^. The diameter of the patch dome at any specific negative hydrostatic pressure was estimated using the ZEN software and was used to calculate the corresponding membrane tension using La Place’s law ^37, 38^.

### Spin-labelling and EPR spectroscopy

The purified MscL proteins were labelled, with the spin label (1-oxyl-2,2,5,5-tetramethylpyrroline-3-methyl) methyl methanethiosulfonate (Toronto Research) at a molar ratio of 10:1 spin label/protein. Proteins were reconstituted into liposomes made of POPC lipids (1-Palmitoyl-2-oleoyl-sn-glycero-3-phosphocholine, Avanti Polar Lipids, Inc., USA) at a 1:2000 protein/lipid molar ratio by dilution in PBS pH 7.5 buffer. As a result, in a fully labelled channel, a spin label attaches to each of the five MscL subunits. EPR spectroscopy was carried out using the EPR spectrometer EMX with the dielectric resonator ER 4119HS (Bruker instrument, Karlsruhe, Germany). The unattenuated magnetic field power was 200mW. Samples were fixed with gas permeable TPX capillaries (L&M EPR supplies, Inc., Wisconsin USA), allowing for the saturation of sample with O_2_ and N_2_. The EPR mobility measurements were obtained from the first derivative spectrum centred on the central peak recorded over a sweep width of 150 Gauss. Power saturation curves were obtained for each spin-labelled mutant after equilibration in N_2_ as control, and air (approximately 20% O_2_), and N_2_ in the presence of 50 mM NiEdda for 15 min at 37°C as relaxing agents. After all closed state measurements had been recorded, lysophosphatidylcholine (LPC) was added to samples to a final molar ratio of 1:3 (LPC: DOPC), which corresponds to 25% LPC, and all measurements were repeated with these LPC samples. All EPR data were obtained at room temperature and analysed with the WINEPR software.

### All-atom Molecular Dynamics Simulations ( Ec MscL WT, Q65E, Q65R)

All systems were built with CHARMM-GUI by embedding *E. coli* MscL (EcMscL) in a mixed lipid bilayer containing ~ 200 lipids total at 70% DOPC / 30% DOPG (≈ 140 DOPC + 60 DOPG). Three protein variants were simulated: WT, Q65E, and Q65R. Mutants were generated in CHARMM-GUI by substituting residue Q65 with E or R, then inserting the resulting protein into the same bilayer composition to form the corresponding mutant systems. Each solvated system contained ~ 230,000 atoms. Bilayers were solvated using TIP3P water with a reported water layer thickness of ~ 22.5 Å (buffer above and below the membrane), yielding a simulation box of approximately 143 × 141 × 151 Å. Systems were neutralized prior to simulation.

Simulations were performed with GPU-accelerated NAMD 3.0 using the CHARMM36 force field. Trajectory visualization and analysis were carried out with VMD software. Equilibration followed the same protocol as in our prior simulations ^13, 39^, and system building followed established CHARMM-GUI workflows. System equilibration was monitored using RMSD and total energy. All systems reached stable behaviour (RMSD plateau and stabilized energy) after approximately 200 ns, which was taken as the equilibration endpoint. Temperature was maintained at 303 K using a Langevin thermostat. Pressure control used Langevin dynamics with a modified Nosé–Hoover barostat. Long-range electrostatics were computed using PME with a 12 Å real-space cutoff and 1 Å Fourier grid spacing.

### In silico force-application (tension/pulling)

After equilibration, channel opening was induced by applying an in-plane, radially outward constant force to the N-terminal domains of EcMscL. The force magnitude was 0.5 kcal/mol·Å (~ 35 pN), chosen to remain below the reported ~ 100 pN unfolding tolerance of MscL helices ^39, 40^. The N-terminus was targeted because the amphipathic N-terminal helix is known to contribute critically to bilayer-coupled gating, stabilizing the closed state and coupling membrane tension to the channel ^12^. In parallel, a uniform in-plane distributed membrane tension of 30 mN/m was applied across the bilayer to reduce suppression of channel deformation by lateral crowding from surrounding lipids. Because the Q65E and Q65R substitutions alter local electrostatics and contacts near the periplasmic vestibule, compared to WT, we expected the same applied mechanical stimulus to produce variant-specific opening kinetics and permeation. Channel opening kinetics were quantified using (i) a pore-diameter proxy defined as the distance between V21 residues on opposing subunits, and (ii) the number of water molecules permeating/occupying the pore region as a measure of hydration and permeability. In this simulation, water molecules were selected within a cylindrical region defined by a radius of 10 Å and a height spanning the space between the periplasmic loop and the C-terminal domain. This geometric selection allowed precise quantification of water penetration through the channel during conformational changes. Structural snapshots were extracted to compare closed vs open conformations across variants.

### Statistical analysis

Statistical analysis was performed using One-way ANOVA analysis of variance together with a Dunnett post hoc test, and significant differences between groups were considered at P < 0.05. The number of replicates is included in the figure legends. The values are presented as mean ± s.e.m., unless otherwise stated.

## Results

### Role of the periplasmic loop in MscL mechanosensitivity

**Figure 1:**
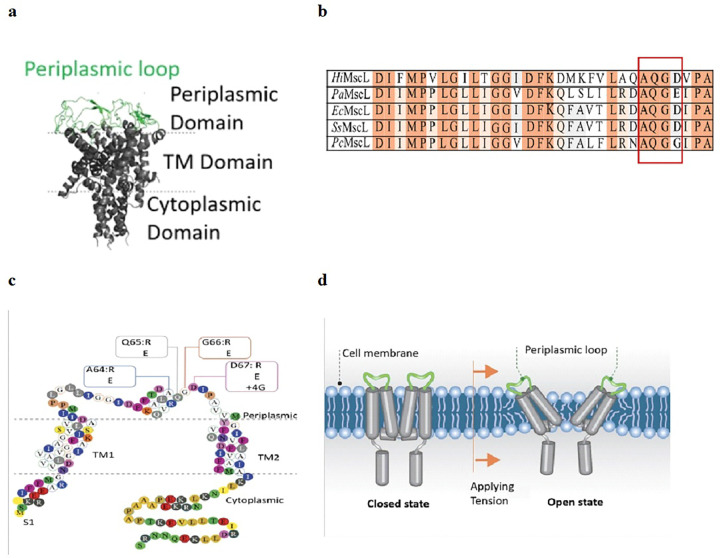
Crystal structure of the MscL protein (PDB: 2OAR) and the sites of periplasmic loop residue substitutions. (a)3D Crystal structure of pentameric TbMscL channel showing the cytoplasmic domain, the two transmembrane domains and the periplasmic loop in a closed state. (b) Sequence alignment of MscL homologues from different bacterial species showing that the A64, Q65, G66 and D67 residues are conserved, including *Hi* (*Haemophilus influenzae*), *Pa (Pectobacterium atrosepticum*), *Ec* (*Escherichia coli*), *Ss* (*Shigella sonnei*), *Pc* (*Pectobacterium carotovorum*), (c) The amino acid sequence of MscL showing the mutations in the periplasmic loop that were examined in this study. (d)A cartoon showing the periplasmic loop of E. *coli (Ec)*MscL in closed and open configurations in response to membrane tension. Note the periplasmic loop being in contact with the lipid bilayer in the open state of the channel.

The *Ec*MscL protein monomer consists of 136 amino acids folded as two transmembrane domains, the cytoplasmic N- and C-terminal domains, and the extracellular periplasmic loop interconnecting the two transmbrane domains. Five monomers combine to form a homopentameric channel (Fig. 1a) which is activated by bilayer tension according to the “force-from-lipids” principle ^10, 5^. Periplasmic loop consisting of 22–29 residues is well conserved between bacterial species (Fig. 1b) and it has been proposed to act as a spring interacting with the phospholipid head groups of the lipid bilayer (Fig. 1d) ^32^.

While the length of the periplasmic loop varies between different bacteria (between 22–29 amino acids) across species, there is a highly conserved region starting from D39, extending to the end of the loop close to the transmembrane domain TM2 with the consensus sequence AQG- [D, E, G] as shown in Fig. 1b. The EcMscL periplasmic loop has a relatively high percentage of charged residues (24%) and a net charge of 0.09 at pH 7.0 (**Table S1**). The substitution of A64 to E or R increased the percentage of charged residues to 28%, and the net charge for these substitutions was − 0.91 and 1.09, respectively (**Table S1**). The mutation of D67 to R also increased the net charge of the loop, whilst the addition of four additional glycine residues (D67 + 4G) at the same position of the loop reduced the percentage of charged residues from 24% to 21% (**Table S1**). As discussed further, the overall charge of the loop is an important determinant of the channel gating properties resulting from electrostatic protein-lipid interactions, as well as determining the significance of each amino acid, gaining a negative or positive charge over the native state at that periplasmic position.

### EPR spectroscopy of the periplasmic loop in EcMscL

The incorporation of a nitroxide spin label to a protein is crucial for SDSL EPR studies. As detailed in the “Materials and Methods”, we adopted the approach described by Perozo et al ^41^ by substituting each residue with cysteine used to attach a nitroxide spin label.

**Figure 2:**
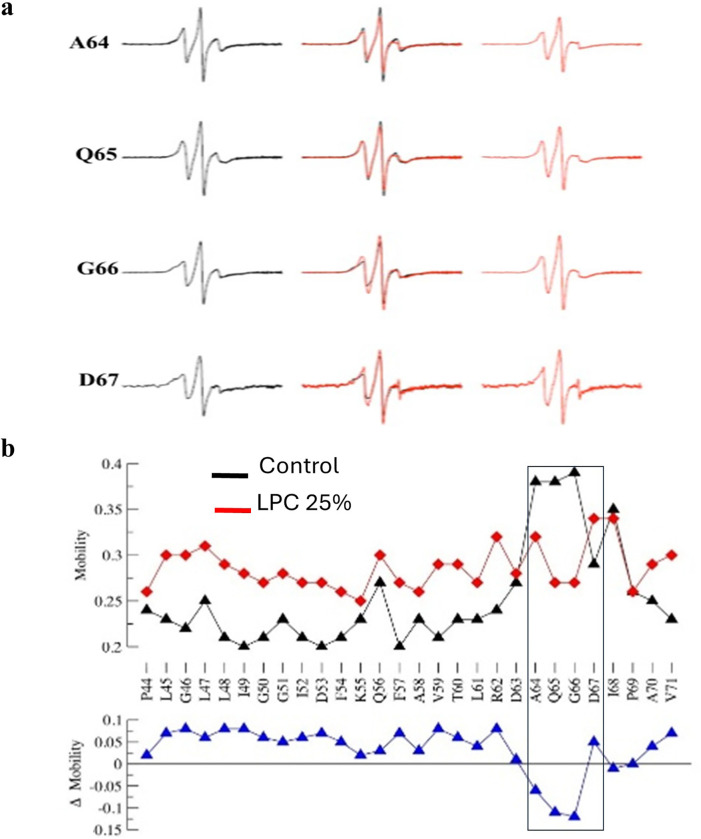
Mobility of the MscL periplasmic loop residues in liposomes probed by EPR spectroscopy. (a) EPR spectra obtained from MscL at different configurations. From left to right; closed state of the channel (black spectra) superimposed spectra of the closed and open channel (middle column), and open state of the channel following the addition of 25% LPC (right column, red spectra). All spectra have been corrected at baseline and normalized to the number of spins. (b) Change in the mobility of the periplasmic residues in the closed and open conformation of the channel (black rectangle showing the periplasmic residues selected for our study): closed state measurements (black triangles) and open state measurements (red triangles). The lower graph (blue) shows the difference in residue mobility between the closed and open states. (Reproduced from 43 with permission)

The mobility of the periplasmic loop residues (Fig. 2) in the closed state has a repeating pattern between residues L47 and Q56. Residues A64 to G66 exhibited very high mobility in the closed state. The values decreased to previous levels towards the end of the loop. The conformational changes of the channel protein were determined from the spectral line shape variations, which correspond to probe dynamics (H_0_). The incorporation of 25% LPC into the lipid bilayer caused changes in the transbilayer pressure profile, thus leading to marked spectral changes ^42^ reflected in the change of the mobility levels recorded for almost all residues. For all but a few residues the mobility increased. The exceptions are residues A64 to D67, which became less mobile, and residues I68 and P69, which did not change. The narrower EPR spectra indicate that the addition of LPC highly favoured the transition to the open configuration of MscL ^42^. Thus, additions of LPC to the liposomes overall enhanced the mobility of the spin label, which is indicated by the sharpening of the EPR spectra (Fig. 2a), except for the spin labelled A64-D67 residues, which exhibited a reduction in their mobility in the presence of LPC (Fig. 2b).

**Figure 3:**
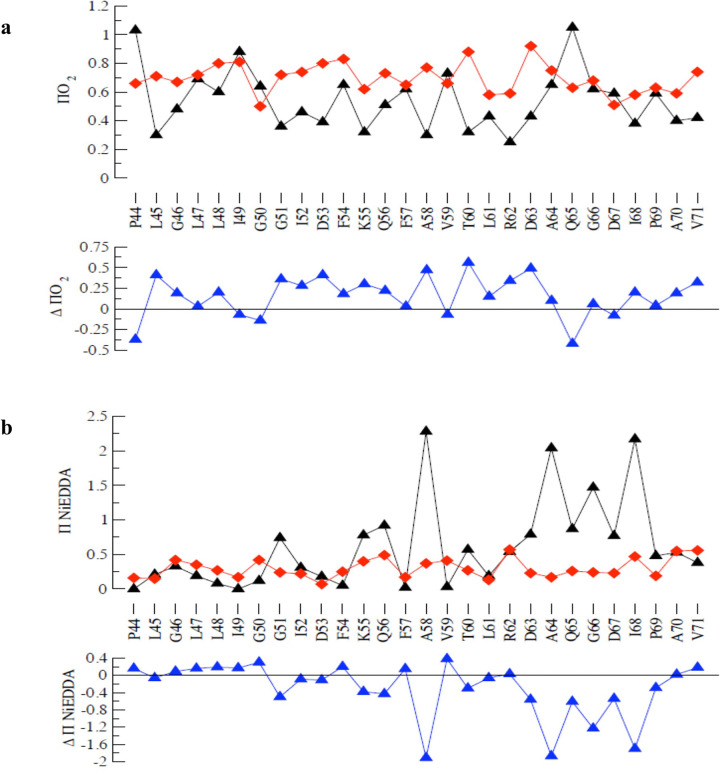
Solvent accessibilities in the presence of O2 and NiEdda. (a) Oxygen accessibility by residue. Shown are the closed state in black (triangles) and open state measurements in red. The lower graph (blue) shows the difference between closed and open states. (b) NiEDDA accessibility by residue. Shown are the closed state in black (triangles) and open state measurements in red. The lower graph (blue) shows the difference between closed and open states. (Reproduced from 43 with permission).

Solvent accessibilities were estimated from power saturation experiments performed in the presence of either atmospheric oxygen or a water-soluble, neutral Ni^2+^ chelate complex (NiEdda) **(Table S2)**. Whereas high accessibility to molecular oxygen O_2_ (ΠO_2_) is indicative of a residue exposed to membrane lipids; residues exposed to the aqueous environment display high NiEdda (Π_NiEdda_) accessibilities ^44^. In the closed state, no detectable periodicity in oxygen accessibility of the periplasmic loop residues was observed (Fig. 3a). Only two peaks at residues I49 and Q65 could be identified. In the open state resulting from the addition of 25% LPC ^42^ a general increase in oxygen accessibility was observed, with the exceptions of residues P44 and Q65, which in addition exhibited a large decrease in O_2_ accessibility. The accessibility to NiEDDA (Fig. 3b) of the periplasmic loop residues in the closed state varied largely for residues between G51 and P69, particularly for the region of high mobility between A64 and D67. The accessibility measurements in the open state after addition of 25% LPC showed no significant change for residues between L45 and G50. The residues D63 to I68 all showed significantly decreased NiEDDA accessibility in close correspondence with the mobility data obtained for these residues (Fig. 2b). For the remaining residues the NiEDDA accessibility was decreasing further, whereas accessibility of residues D53 to L61 exhibited a periodic motif.

### Substitution of the EcMscL periplasmic loop residues alters the channel gating properties

**Figure 4:**
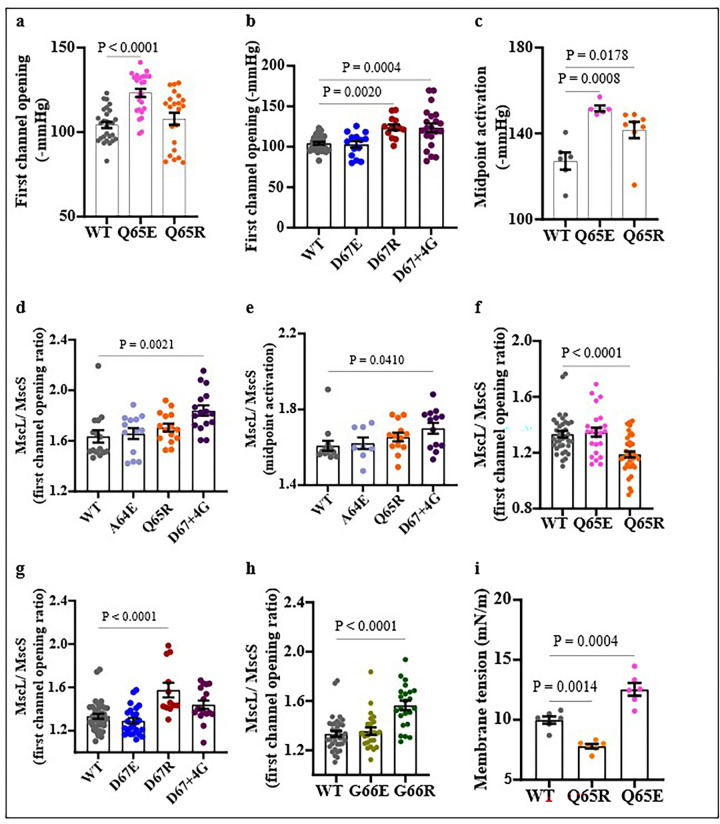
Effect of the periplasmic residue mutations on the activation of *Ec*MscL channel in giant spheroplasts and azolectin liposomes recorded at pipette voltage V_p_ = +30mV. (a-c) Comparison of the first opening and midpoint activation of MscL mutant channels expressed alone in *E. coli strain* MJF612: (a) The substitution of Q65 to E but not R decreased the pressure sensitivity of the channel, thus Q65E mutant required higher pressure for its first opening. (b) The substitution of D67 to R or addition of 4 glycines at the same position reduced the pressure sensitivity of the channel, making it harder to open compared to the WT MscL channel. (c) The substitution of Q65 to either E or R decreased the pressure sensitivity of the channel, thus Q65E and Q65R required higher pressure for the midpoint activation of the channel (d-e) Effect of periplasmic residue mutations on the first opening and midpoint activation of MscL and MscS expressed in *E. coli* AW737KO strain: (d&e) The addition of 4 glycines to MscL periplasmic residue D67 decreased the sensitivity of the channel, leading to channel opening at a higher pressure, both first opening and midpoint activation of the channel, compared to WT MscL. (f-h) Comparison of the ratio of the first opening of MscL and MscS co-reconstituted in azolectin liposomes: (f) Replacing Q65 with R increased the pressure sensitivity of the channel, leading to the mutant channel opening at a lower pressure compared to WT MscL (Fig S2). (g, h) Mutating residues G66 and D67 to R decreased the pressure sensitivity of the channel. (i) Comparison of the membrane tension required for the first opening of mutant and WT MscL reconstituted in 99.9 % azolectin + 0.1% rhodamine PE liposomes using patch fluorometry. Substitution of Q65 to R lowered membrane tension required for activation of the channel, while replacing the same MscL periplasmic residue with E increased the tension required for the first opening of the channel. Data are presented as mean ± s.e.m., one-way ANOVA. (number of replicates n >10 for each MscL mutant).

Previously, two amino acids (Q51 and Q65) in the periplasmic loop have been substituted and shown to contribute to the gating properties of the channel ^32, 45^. It remains, however unclear if all the periplasmic loop residues or just a few key residues were crucial for regulating the mechanosensitive gating of the channel. Our study shows that Q65 is crucial for the gating of MscL and that mutations to either a positively charged R or negatively charged E (as highlighted in **Table S1**) affect the gating properties of the channel (Fig. 4). Using patch fluorometry ^37, 38^ and Laplace’s law, we estimated the membrane tension (**Fig. S1**) required for the first opening of the channel and found that Q65R requires lower membrane tension (7.80 ± 0.19 pN/nm) compared to the wild-type MscL (12.54 ± 0.52 mN/m), whilst Q65E opens at a higher membrane tension (9.98 ± 0.30 pN/nm) (Fig. 4 &. **S1**). The insertion of a hinge region (D67 + 4G) between the periplasmic loop to TM2 decreased the sensitivity of the channel, making it harder to open (opening pressure: −123.4 ± 5.4 mmHg) compared to WT MscL (−104.2 ± 1.8 mmHg) when expressed in *E. coli* strain MJF612 (Fig. 4b). For a relative reference value, the MscL channels under study were co-expressed with MscS in *E. coli* AW737KO strain, and the ratio of the pressure required for activation of both MscS and each MscL mutant in the giant spheroplast was calculated (Fig. 4d & e, **S5**). As presented in Fig. 4d & e, the addition of 4G residues to position D67 resulted in higher MscL: MscS ratio for the first opening (1.84 ± 0.04) and midpoint activation (1.70 ± 0.03), compared to the wild-type MscL channel (first opening ratio: 1.64 ± 0.05; midpoint activation ratio: 1.61 ± 0.03). Substitution of the periplasmic residues G66 and D67 to R reduced the pressure sensitivity of the channel as well, thus leading to an increased MscL: MscS ratio for the first opening in azolectin liposomes compared to WT MscL (WT: 1.33 ± 0.02; G66R: 1.56 ± 0.04; D67R: 1.58 ± 0.07). Of the four periplasmic residues that were mutated in our study, mutating A64 was found to have the least effect on the channel gating, as substituting it with E or R did not affect the channel activation by membrane tension.

### MscL periplasmic loop plays significant role for the channel opening kinetics in negatively charged lipids

**Figure 5:**
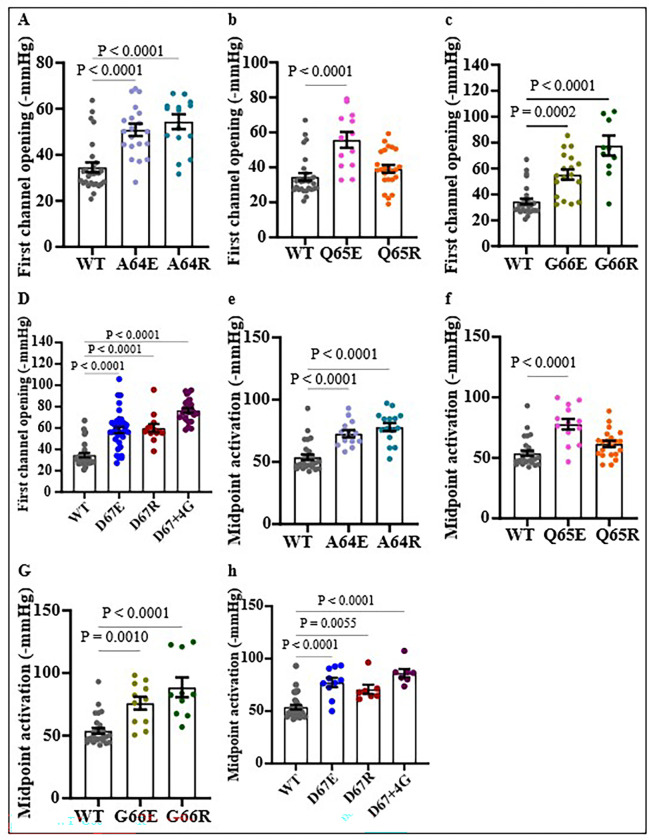
Functional studies showing the effect of the periplasmic residue mutants in a negatively charged lipid bilayer. Comparison between the first (a-d) and midpoint openings (e-h) of MscL mutants in negatively charged lipids (DOPC 70 % and DOPG 30%). Mutation of A64, G66, and D67 to E or R decreased pressure sensitivity of the channel as shown for both the first opening (a-c)and midpoint activation of the channels (f-h). Addition of 4 glycines at D67 site reduced the first opening and midpoint activation of the channels (c & h). Substitution of Q65 to R did not affect the first opening and midpoint activation (d&e), but mutation to E decreased the tension sensitivity of the channel, requiring higher pressure for both the first opening (d) and midpoint channel activation (h).

The effect of various lipids on MscL gating has been widely studied showing that the WT MscL channel gating remains unaffected by the presence of the anionic lipids phosphatidylserine (PS) or phosphatidylglycerol (PG) but opens at a higher membrane tension in bilayers made of phosphatidylethanolamine (PE) than in bilayers made of phosphatidylcholine (PC)^46^. Since lipid composition can affect the gating of MscL, we examined how periplasmic loop residues contribute to the opening of the mutant channels in negatively charged liposomes comprising of 70% PC and 30% PG lipids. All our periplasmic mutations except Q65R led to a decrease in the pressure sensitivity of the channel (Fig. 5). For example, we found that mutations in the A64 periplasmic residue influenced the gating of MscL channel only in negatively charged lipids. Substitution of A64 to E or R decreased the pressure sensitivity of the channel, making it harder for the channel to open. The midpoint activation pressure for A64E (−72.7 ± 2.9 mmHg; p < 0.0001) and A64R (−78.1 ± 3.2 mmHg, p < 0.0001) is significantly higher compared to WT MscL (−53.8 ± 2.3 mmHg). Interestingly, substitution of negatively charged D67 with functionally equivalent E (D67E) decreased the pressure sensitivity of the channel (D67E: −74.2 ± 5.8 vs WT: −53.8 ± 2.3 mmHg). Also, the midpoints of the Boltzmann distribution functions of opening probabilities of the MscL mutants and WT channel **(Fig. S5)** plotted against negative hydrostatic pressure applied to patch pipettes were right shifted in corresponding manner on the pressure axis **(Fig. S3).**

### MscL WT and mutant channel opening kinetics examined by MD simulations

**Figure 6:**
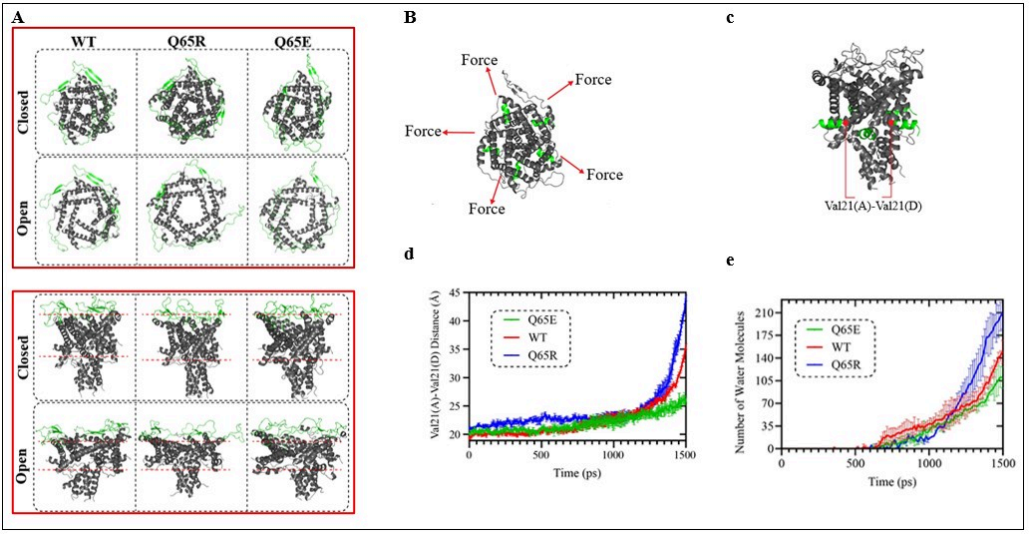
Structural snapshots of EcMscL gating under tension. (a) Top and side view (bottom plane) of WT, Q65R, and Q65E channels in representative closed and open states under applied mechanical stress (constant-force pulling plus membrane tension). Gating kinetics from force-application simulations: (b–e) After equilibration, WT, Q65E, and Q65R EcMscL were subjected to constant-force pulling using a 35 pN radial in-plane force applied to the N-terminal domains, together with a uniform membrane tension of 30 mN/m (b–c). Channel opening was quantified by pore diameter (defined as the distance between V21 residues on opposing subunits) and by pore hydration/permeation (d–e).WT and Q65R exhibit earlier and larger increases in pore diameter than Q65E, consistent with a lower activation threshold and highlighting the functional importance of residue 65 in gating; representative snapshots illustrate variant-dependent conformational rearrangements during opening (d). Water occupancy within the pore region increases with opening, with Q65R showing the highest hydration/permeation (approximately 210 waters in the final frames), WT intermediate (approximately 150), and Q65E the lowest, consistent with reduced opening (d). Each simulation was repeated five times, and data represented in d and e are presented as mean ± s.e.m.

Using molecular dynamics (MD) simulations, we investigated the tension-induced gating of the MscL WT and mutant (Q65R and Q65E) channels (Fig. 6 & **S4**) to assess the force and time required to change the radius of the central pore during opening. All three channel types (WT, Q65E, Q65R) reached stable RMSD plateaus during equilibration, supporting the use of the equilibrated structures as starting points for the tension/pulling simulations (**Fig S4**). Notably, the mutations did not appreciably alter bilayer thickness prior to tension application. Under identical pulling force and membrane-tension conditions, the applied stimuli were sufficient to drive each construct from a closed to an open-like conformation (Fig. 6 **and SI Movie 1**). Despite this common closed-to-open transition in all the models, the variants exhibited clearly distinct opening kinetics, consistent with experimental observations showing differences in their gating threshold and activation behaviour (Fig. 6). In the closed state, Q65E displayed subtly loosened periplasmic packing and underwent a more uniform progression in the channel conformational change during opening. In contrast, WT and Q65R retained tighter packing prior to strong deformation when undergoing a more abrupt, cooperative expansion characterized by pronounced outward displacement of the helices. Q65E showed the smallest degree of pore expansion (Fig. 2d) and consequently the lowest water permeation (Fig. 2e). WT displayed an intermediate response, reaching a maximum of approximately 180 water molecules in the pore in the final phase. Q65R underwent the largest expansion and exhibited the highest water permeation, reaching approximately 220 water molecules in the final phase (Fig. 2d & e). These *in silico* trends are consistent with the experimental interpretation that electrostatics and local residue interactions tune the energetic barrier for activation and shape gating kinetics. Together, the results indicate that the electrostatic environment at the periplasmic loops, centred on the 65 residue, strongly modulates both the opening threshold and the kinetics of the channel opening.

## Discussion

MscL is fundamentally important for bacterial osmoregulation, serving as an emergency pressure-release valve ^47^. Beyond this essential biological role, MscL has long been established as the prototype mechanosensitive channel for studying fundamental lipid-protein interactions that underpin mechanotransduction across all kingdoms of life ^48^. Its simple structure and clear function have also led to the proposal by many groups for its advanced applications, including the use as a nanovalve, ultrasound-activated mechanoactuator, or tension gauge in mammalian systems ^49, 50, 51^. Although MscL is arguably the most extensively characterized mechanosensitive channel, the contribution of its periplasmic loop remains surprisingly vague; this structural segment connects transmembrane helix 1 (TM1, the pore-forming helix) with transmembrane helix 2 (TM2, the lipid-facing helix).

Extracellular loops are frequently present in bacterial and human MS channels and have been suggested to play a role in mechanosensitivity of these channels ^52, 53, 35, 32^. Mutation of glycine 168 to glutamate (G168E) or the substitution of isoleucine 462 to alanine (I462A) in the extracellular loop of the human TMEM63A mechanosensitive cation channel was found to prevent stretch-activated currents, causing infantile-onset of transient hypomyelination ^52, 54, 53^. Previous MscL studies that were primarily focused on substituting Q56 and Q65 residues of the periplasmic loop, indicated that the periplasmic loop plays critical role in the mechanosensitivity of the channel ^28, 35^. Following our EPR spectroscopy data ^43^, we observed that in addition to Q65, periplasmic residues A64, G66 and D67 exhibited increased change in mobility in the closed and reduced mobility in the open channel state, respectively, where the channel opening was induced by the addition of LPC to one monolayer of the bilayer, which caused a change in the local bilayer curvature and thus activating the channels ^55^ (Fig. 2b).

Previous studies that investigated the contributions of the periplasmic residues to the mechanosensitivity of MscL were mainly evaluated based on sensitivity of the channels to hydrostatic pressure ^32, 35, 28^. However, this approach did not account for patch geometry which influences the bilayer tension required for the activation of these mutant channels ^37^. Hence, in our study, we designed well-controlled experiments to understand how a group of periplasmic residues (A64, Q65, G66 and D67) influence mechanosensitivity of the *E coli* MscL by using different approaches, including estimating the membrane tension required for opening of the channels and using the pressure ratios of MscL to MscS for the first and midpoint activation in giant spheroplasts and proteoliposomes **(Fig. S5)**. Previous work demonstrated that substituting K101 at the TM2-cytoplasmic domain of *Ec*-MscL channel to negatively charged aspartate (D) or glutamate (E) decreased the channel sensitivity to membrane tension suggesting that charge interactions between the protein and the lipid bilayer plays significant role in the mechanosensitivity of the channel ^49^. Based on this, we explored how charge substitution at these periplasmic residues affects the biophysical properties of MscL in different lipid environments using functional patch clamp and fluorescence patch clamp techniques as well as molecular dynamics simulation.

Further to studying the biophysical properties of the MscL mutant channels in azolectin liposomes, we also investigated how these residues might affect the mechanosensitivity of MscL in a well-defined negatively charged liposomes comprising PC 70% and PG 30%, which is an artificial system that allows us to mimic the *E. coli* cell membrane. Previous work showed that bacterial membranes are composed of various lipids, especially phosphatidylethanolamine (PE) and phosphatidylglycerol (PG), providing an overall negatively charged lipid environment that is essential for normal function and osmoregulation in bacterial cells ^14, 56, 49^.

We also investigated how lipid bilayer thickness and tension could affect the opening of WT MscL, Q65R and Q65E using molecular dynamic simulations in negatively charged PG lipids (Fig. S4). Previous reports showed that substitution of Glutamine (Q) 65 to Arginine (R) increased the pressure sensitivity of the channel ^35, 57^. Our findings further confirmed that Q65R mutation increased the pressure sensitivity of the channel corresponding to reduced membrane tension required for the activation of the channel in azolectin liposomes (Fig. 4, **Fig. S1 & S2**). However, substitution Q65R showed an opposite effect in giant spheroplast by decreasing the pressure sensitivity of the channel. Q65R thus required higher pressure for the midpoint activation of the channel (Fig. 4c). Although we observed that in negatively charged proteoliposomes the pressure required for the opening of Q65R was not different compared to WT MscL (Fig. 5), our molecular dynamics simulations data suggest that Q65R requires less force to open the channel in a negatively charged lipid bilayer. Interestingly, our molecular dynamics stimulation data mimics patch-clamp recordings of Q65R activities in azolectin liposomes (Fig. 6) despite the difference in the lipid environment. Investigating this discrepancy is presently out of scope of this study and will be addressed in our future work.

Our study also found that replacing Q65 with glutamic acid (E) reduced the channel’s sensitivity to membrane tension in both azolectin (Fig. 4e) and negatively charged liposomes, as well as in giant spheroplasts (Fig. 4a, 5a, b). This finding is further supported by our molecular dynamics simulation data (Fig. 5c, d). Substituting the periplasmic residue A64 to either glutamic acid (A64E) or arginine (A64R) or replacing G66 with glutamic acid (G66E) did not significantly affect mechanosensitivity of the channel in azolectin liposomes and giant spheroplasts, although these mutations decreased the channel’s tension sensitivity in negatively charged liposomes. Insertion of a flexible “decoupling” sequence of residues by addition of four glycines (4G) after D67 residue in the periplasmic loop decreased pressure sensitivity in giant spheroplasts, azolectin, and in negatively charged liposomes (PC70:PG30). We suggest that adding flexibility into the periplasmic loop by 4G disrupts the transmission of mechanical force from “the slack periplasmic loop” to the pore-lining helix, thereby decreasing channel sensitivity. This interpretation is consistent with a previous study reporting that insertion of two or five glycines at the G14 site in the N-terminal domain of MscL impaired transmission of mechanical force from the N-terminus to the TM1 pore-lining helix, thus reducing pressure sensitivity ^12^.

Furthermore, AlphaFold prediction of the periplasmic loop structure of EcMscL suggests the presence of a beta-hairpin formed by the first strand from Val59 to Gln65, Gly66-Asp67 loop and the opposite strand from Ile68 to Met73 **(Fig S6)**. This beta-hairpin may be responsible for the suggested role of the periplasmic loop in setting the energy barrier required for the opening of MscL ^32^. Based on the AlphaFold predictions, our mutation sites lie around the beta-hairpin region which might break or introduce additional hydrogen bond(s) at the periplasmic loop, which would influence how the periplasmic loop interacts with different lipid environments and gating mechanism of the channel **(Fig S6)**. For example, Q65E mutation which may add an extra inter subunit hydrogen bond between Arg62 side-chain eta(η)-nitrogen and Gly50 within an adjacent subunit of the periplasmic loop, could cause a decrease in pressure sensitivity of the channel, while Q65R, which results in the loss of an intra-strand hydrogen bond (between Gln65side-chain oxygen to Arg62 side-chain eta(η)-nitrogen) would lead to increased sensitivity of the channel **(Fig S6)** in agreement with the molecular dynamics simulations and results of the patch-clamp recording from the mutants reconstituted into azolectin liposomes (Fig. 4i). Overall, our findings suggest that the impact of the periplasmic loop mutations on MscL mechanosensitivity largely depends on electrostatic interactions between a few periplasmic residues and their lipid environment ^49^.

## Conclusion

Although this seems not to be the case for all different types of mechanosensitive channels, the structure of some of those channels contains an extracellular loop that controls their gating properties. Our study provides definitive spectroscopic, electrophysiological and computational evidence for the periplasmic loop of *E. coli* MscL as a major contributor to the channel activation by mechanical force not only by adjusting the channel sensitivity to membrane tension but also to modulation of its kinetics of conformational changes underlying opening of the channel pore resulting from protein-lipid electrostatic interactions. We suggest that our finding showing that MscL periplasmic loop functions as a regulator of the channel opening kinetics represents a novel common principle underlying the channel gating of unrelated MS ion channels, which possess functionally important extracellular loops. This finding has several implications, including understanding of (i) the cells’ ability to exhibit a more nuanced, graded response to varying degrees of mechanical tension, (ii) the specialization of MS channels for specific physiological roles, (iii) specific protein-lipid interactions that could reveal novel targets for therapeutic interventions, as well as (iv) how the same MS channel type behaves differently depending on its cellular location or the cell's physiological state, which adds another layer of regulatory complexity to mechanosensation.

## Supplementary Material

Supplementary Files

This is a list of supplementary files associated with this preprint. Click to download.

• Duruetal.SUPPLEMENTARYfile.docx

## Data Availability

All data generated or analysed during this study are included in this published article [and its supplementary information files].
